# Blood Pressure Management in Acute Ischemic Stroke With Concurrent Intracranial Neoplasm and Intratumoral Hemorrhage

**DOI:** 10.7759/cureus.68045

**Published:** 2024-08-28

**Authors:** Beatriz Castro Silva, Miguel Serôdio, João Ramos

**Affiliations:** 1 Internal Medicine, Hospital Beatriz Ângelo, Loures, PRT; 2 Neurology, Centro Hospitalar Lisboa Ocidental, Lisbon, PRT; 3 Neuroradiology, Centro Hospitalar Lisboa Ocidental, Lisbon, PRT

**Keywords:** schwannomma, complication of treatment, stroke treatment, cerebro-vascular accident (stroke), haemorragic schwannoma

## Abstract

Acute ischemic stroke is a major cause of morbidity and mortality worldwide. Furthermore, careful clinical assessment combined with neuroimaging is crucial for an accurate diagnosis and allows other differential diagnoses to be determined. We report a case of an 81-year-old female with a history of hypertension who presented with dysarthria, left central facial paresis, and right oculocephalic deviation. Cranial CT revealed no acute ischemic lesions, and no vessel occlusion was detected in CT angiography. Incidentally, an extra-axial left lateropontine space-occupying lesion with recent bleeding was detected. The patient remained under surveillance with permissive hypertension, without antithrombotic drugs. A clinical worsening with somnolence and left VI and VII nerve palsies followed, motivating cranial CT repetition, which disclosed aggravated bleeding of the space-occupying lesion and an acute right frontal ischemic lesion. Upon discussion with Neurosurgery, no surgery was offered, and the patient was admitted to the Stroke Unit, with strict blood pressure control and delay of antiplatelet initiation. At the time of discharge, the patient showed neurological improvement.

Permissive blood pressure regimens in patients with acute ischemic stroke not reperfused are still not well studied when concurrent intracranial tumors exist, where the potential to aggravate/precipitate intratumoral hemorrhage exists. This case report highlights the need to better delineate the strategy regarding blood pressure control in these patients.

## Introduction

Stroke is a condition characterized by a high prevalence and mortality rate [1,2], emphasizing the critical role of clinical evaluation in conjunction with neuroimaging for an accurate diagnosis. Moreover, a thorough examination of the neuroimaging facilitates the identification of potential alternative diagnoses. In the acute phase of ischemic stroke, blood pressure targets are well-defined [1,2]. However, there is limited guidance when concomitant pathologies, such as intracranial tumors with a risk of hemorrhage, are present.

## Case presentation

**Figure 1:**
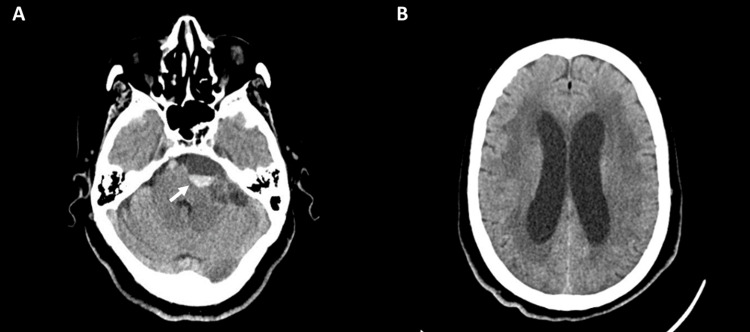
(A,B) Axial cranial CT revealing extra-axial space-occupying lesion (arrow) in the left angle-cerebellar cistern without other lesions.

**Figure 2:**
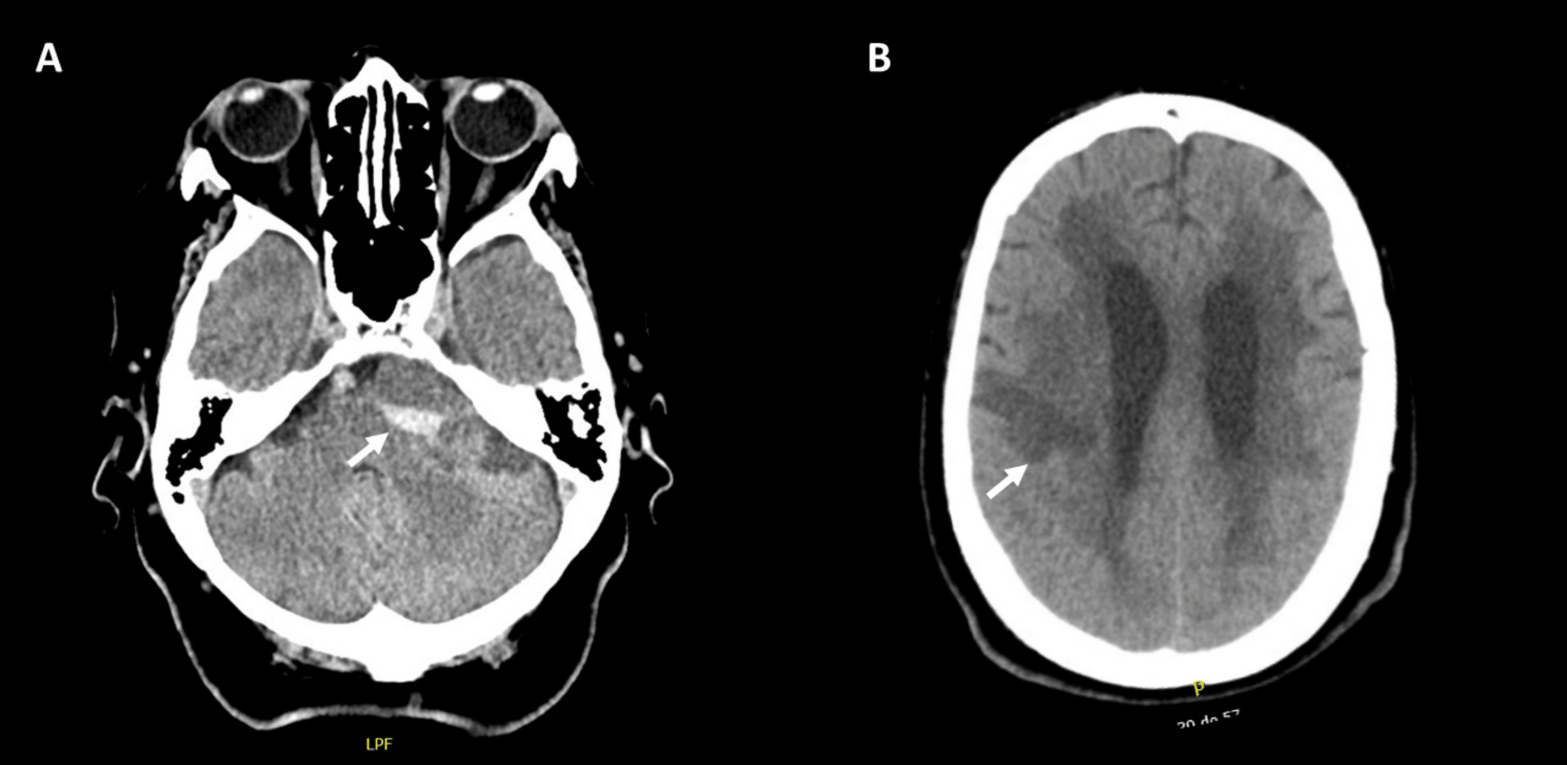
(A,B) Axial cranial CT documenting increased hematic density of the space-occupying lesion (arrow in A), without obstruction of the ventricular system, and recent ischemic lesion in the partial territory of the right middle cerebral artery (arrow in B).

**Figure 3:**
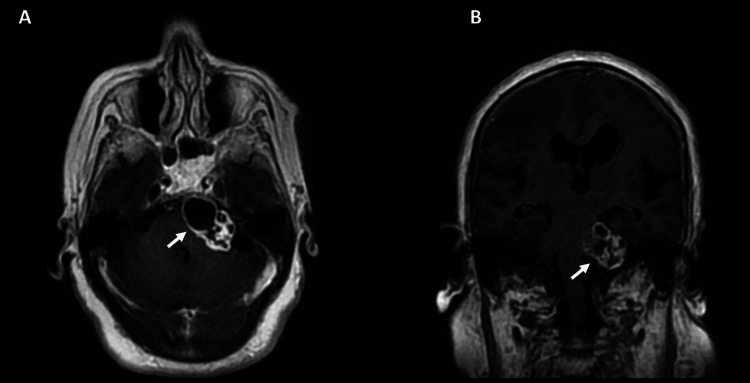
Post-gadolinium T1 in the (A) axial and (B) coronal planes demonstrating extra-axial space-occupying lesion in the cistern of the left cerebellopontine angle with a necrotic-cystic-hemorrhagic appearance with peripheral enhancement (arrows).

We report a case of an 81-year-old female with dyslipidemia and hypertension who presented to the emergency room with a sudden onset of dysarthria, left central facial paresis, and forced oculocephalic deviation to the right, with four hours of evolution. No mental status, hemianopia, and limb deficits were present. Upon admission, her blood pressure was 240/120 mmHg. Cranial CT (Figure 1) did not show ischemic lesions, and no vessel occlusion was visible on CT angiography. Incidentally, an extra-axial left lateropontine space-occupying lesion (SOL) with recent bleeding was present. The patient remained under surveillance with permissive hypertension, and no acute reperfusion treatment nor antithrombotics were offered (no vessel occlusion, presence of intracranial hemorrhage). Upon reassessment, three hours later, the patient worsened with drowsiness, paresis of abduction of the left eye, left peripheric facial paresis, and left upper limb paresis. A repeat cranial CT (Figure 2), 30 minutes after new neurological deficits, disclosed increased hematic density of the SOL and an acute right middle frontal ischemic lesion. After discussion with the Neurosurgery team, the decision was made to defer surgery due to the presence of an acute ischemic stroke (AIS). The patient was admitted to the Stroke Unit. Due to the intratumoral hemorrhage worsening, conservative measures with blood pressure at a target of <140/90 mmHg were initiated, and antiplatelet therapy was postponed. A brain magnetic resonance imaging (Figure 3) showed an extra-axial SOL in the left cerebellopontine angle, which most probably constituted a vestibular malignant schwannoma with necrotic-cystic and hemorrhagic components. Regarding stroke etiology, no cause was identified following a comprehensive evaluation, including blood tests for vascular risk factors, supra-aortic vessel Doppler ultrasound, transthoracic echocardiography, and prolonged cardiac rhythm monitoring. The patient showed neurological improvement until the time of discharge, without the presence of ataxia or deficits of the VIII or V cranial pairs. Single antiplatelet therapy was initiated after a controlled cranial CT revealed no additional hemorrhage. This was accompanied by an increased statin dosage and adjustments to antihypertensive medication. The treatment presented a challenging risk-benefit balance, but antiplatelet therapy was commenced, and the schwannoma was subsequently resected.

## Discussion

Blood pressure management in AIS

The optimal management of blood pressure in AIS and acute intracerebral hemorrhage (ICH) is a subject of ongoing debate [1,3]. For patients with AIS who are not treated with intravenous thrombolysis or mechanical thrombectomy, a cautious approach to blood pressure reduction (<15% systolic blood pressure reduction in 24 hours) is deemed reasonable and likely to be safe, recommended to control blood pressure early and moderately, aiming for a blood pressure of >220/120 mm Hg [1,3]. Both the current European guidelines [1] and the American Heart Association (AHA)/American Stroke Association (ASA) guidelines [3] advise against aggressive blood pressure lowering in most patients during the initial 24 hours unless blood pressure levels are extremely high or there is a specific situation requiring rapid reduction. Acute hypertension in collateral circulation plays a role in maintaining perfusion to brain tissue during ischemic stroke, which prolongs the time window for effective therapies to be beneficial and ultimately avoids irreversible damage that may lead to worse clinical outcomes [4].

Blood pressure management in ICH

On the other hand, in acute ICH, where rapid and intensive blood pressure lowering is recommended immediately after hospital admission to improve recovery by reducing hematoma expansion, treatment should ideally commence within two hours of symptom onset. Because elevated blood pressure is common after the onset of intracerebral hemorrhage and is strongly associated with a poor outcome, a central component of the management of patients is to provide treatment to lower blood pressure towards a systolic target of 140 mmHg or less. [1,5-7].

Vestibular schwannomas and hemorrhage

The cause of bleeding associated with intracranial tumors is connected to various factors that can initiate bleeding, and these factors may originate either locally or systemically [8]. The exact mechanisms of hemorrhage within the brain tumor are unclear, but abnormalities in tumor vascularization seem to play the most important role in etiopathogenesis [8].

Vestibular schwannomas are the most common cerebellopontine angle tumors (±8% of all intracranial tumors and ±85% of all tumors in this location) [9,10]. They are rarely complicated by hemorrhage in 1% of cases [1-3], and hypertension may correlate with intratumoral hemorrhage in vestibular schwannomas [11,12]. However, there are other contributing factors to hemorrhage, such as the size, histopathological vascular architecture of the schwannoma, or the patient's age [10-12].

## Conclusions

The definition of more restricted blood pressure targets could be an important factor in cases of ischemic stroke with pathology that poses a high hemorrhagic risk. The permissive control of blood pressure in patients with ischemic stroke and intracranial tumor is not yet well studied. Due to the potential for intracranial hemorrhage, there may be a need for greater blood pressure restriction. However, this must be balanced against the risk of exacerbating the ischemic lesion, especially in patients with ischemic stroke and untreated vessel occlusion, which can lead to collateral failure.
